# Can Psychopathy Be Adaptive at Work? Development and Application of a Work Focused Self- and Other-Report Measure of the Triarchic Psychopathy Model

**DOI:** 10.3390/ijerph17113938

**Published:** 2020-06-02

**Authors:** Anna Sutton, Maree Roche, Madeleine Stapleton, Anja Roemer

**Affiliations:** 1 School of Psychology, University of Waikato, Hamilton 3240, New Zealand; mbs12@students.waikato.ac.nz (M.S.); ar180@students.waikato.ac.nz (A.R.); 2 Management School, University of Waikato, Hamilton 3240, New Zealand; maree.roche@waikato.ac.nz

**Keywords:** psychopathy, triarchic model, wellbeing, JD-R, leadership

## Abstract

Psychopathy may have both adaptive and maladaptive effects at work but research into workplace psychopathy is constrained by the lack of short, work-relevant measures that can be used for both self- and other-report. We adapt the Triarchic Psychopathy Measure (TriPM) for this purpose and distinguish the (mal)adaptive effects of psychopathy at work in two time-lagged survey samples. Sample 1 consisted of managers reporting their psychopathic traits and work outcomes (well-being, engagement, burnout and job performance). Sample 2 reported on their managers’ psychopathic traits and leadership styles (servant and abusive supervision) and their own work outcomes. The TriPM (Work) is a reliable, valid, 21-item measure of triarchic psychopathy at work with self- and other-report forms. Using this measure, we demonstrate that the triarchic model’s boldness trait is related to servant leadership and predicts improved well-being and performance while meanness and disinhibition are related to abusive supervision and predict increased burnout.

## 1. Introduction

“Psychopath” is a term used by the layperson to refer to a wide range of people, from intractable criminals to businesspeople who prioritise profits over morality, but the underlying concern is that psychopaths cause harm to others [1]. It is this concern that psychopaths are harming workers, their organisations and even the economy and the planet that is behind the recent upsurge in interest in the so-called “corporate psychopath”, who displays many of the traits of psychopathy but not the level of antisocial or law-breaking behaviour that would result in a criminal record [2]. This criminal behaviour was one of the hallmarks of early definitions of psychopathy, but more recently psychopathy has been defined as a pathological condition consisting of enduring deviant behaviour in combination with emotional detachment [3].

While there remains debate in the literature over how to conceptualise psychopathy, the triarchic model is particularly relevant for use in community samples as it avoids assessment of criminal behaviour and evaluates a combination of three traits: boldness, meanness and disinhibition [4]. The recognition that psychopathy involves deviant but not necessarily criminal behaviour has led to the suggestion that some people are able to use their psychopathic traits adaptively in the workplace [5]. In the triarchic model, boldness might represent the adaptive elements of psychopathy while meanness and disinhibition are more maladaptive [5]. However, distinguishing these adaptive and maladaptive effects of psychopathy at work is currently hindered by the lack of an occupationally relevant measure of psychopathy. In this paper, therefore, we develop a measure of psychopathy at work based on the triarchic model of psychopathy, with equivalent self- and other-report versions, and demonstrate its utility in distinguishing between the adaptive and maladaptive effects of psychopathic traits at work.

### 1.1. Psychopathy at Work

The concept of psychopathy originated in the clinical and forensic literature but interest in its prevalence and impact in the workplace has grown quickly. It was recognised that traits associated with psychopathy could potentially be successful in the workplace for several reasons. First, some aspects of psychopathy, such as charm and confidence, are beneficial in the social environment of work [6]. Second, leaders who are fearless and bold can be advantageous both for their followers and the organisation [7]. Third, there are certain occupations where some of the traits associated with psychopathy, such as fearlessness and low reactivity to stress could be extremely beneficial, such as in the military or police [8].

The complex effects of psychopathy are reflected in the conflicting findings of research on work outcomes too. For example, while overall job performance decreases with higher levels of psychopathy, there are positive associations with specific elements of performance such as communication and creativity [9]. In addition, there is evidence that senior managers report significant levels of psychopathy [10], a strong indication that psychopathic traits are in some way adaptive in the workplace, at the very least in helping individuals gain promotion.

In attempting to understand the complexity of psychopathy at work, one of the earliest case studies distinguished between primary and secondary psychopathy, suggesting that “corporate” psychopathy included the primary callous emotional traits but not the anti-social or criminal tendencies of secondary psychopathy [2,6]. Nevertheless, while there is broad consensus on the multi-dimensionality of the psychopathy construct, the number of dimensions and the centrality of each to the construct are still under debate [5].

The triarchic model of psychopathy was developed in an effort to clarify and reconcile these differing conceptions of psychopathy in the literature [11]. It models psychopathy in terms of three distinguishable traits: boldness (indexing confidence, social assertiveness, emotional resilience and fearlessness), meanness (measuring a lack of empathy and capacity for affiliation, as well as contempt for others and a tendency to exploit or be cruel towards them) and disinhibition (including impulsivity and lack of restraint along with hostility and mistrust towards others) [4]. Each of the three traits makes distinct contributions to externalizing psychopathy [12], and the model is particularly appropriate for use in workplace samples as it enables psychopathic traits to be measured as continuous dimensions in the normal population.

The boldness trait may be particularly relevant for understanding the adaptive effects of psychopathy, while the maladaptive and harmful effects of psychopathy are likely to be more strongly related to the meanness and disinhibition traits [5,13]. Boldness, for example, is associated with higher social status and better personality functioning while disinhibition is related to lower status, and both meanness and disinhibition are related to poorer personality functioning [14].

Because boldness may relate to potentially adaptive behaviours [11], some researchers have disputed the relevance of this trait to psychopathy. However, a recent meta-analytic review confirmed that the three triarchic constructs are of equal relevance [15]. Assessing psychopathy with measures that index all three constructs to the same degree is therefore essential, as failing to do so would provide an incomplete picture of how psychopathy manifests [13]. It is surprising then that many studies in the organisational literature have typically utilised measures that report a total psychopathy score or have not reported findings related to each psychopathy factor [16], and this may indicate a need for an organisationally relevant measure of psychopathic traits. There is consensus that psychopathy is an underlying factor in deviant interpersonal behaviours that cause distress for co-workers [17], and there have been several and repeated calls for measures of psychopathy suitable for use in organisational settings [18,19].

The most commonly used measure of psychopathy is the Psychopathy Checklist (PCL) and its various revisions and adaptations [1], which assess psychopathy on interpersonal, affective, lifestyle and antisocial dimensions. The most significant of these adaptations in workplace settings is the B-Scan 360 [18], which assesses the conceptually similar dimensions of Manipulative/Unethical, Callous/Insensitive, Unreliable/Unfocused and Intimidating/Aggressive. The B-Scan is planned as a 360-degree instrument, although to date only the other-rating version has been presented and does not evaluate the boldness element of psychopathy adequately. Another widely used measure of psychopathy, the PPI-R (Psychopathic Personality Inventory-Revised), is a self-report measure developed specifically for community samples and includes subscales to capture the adaptive features of psychopathy such as stress immunity [20]. This measure, however, is fairly long, consisting of 154 items and does not have an equivalent other-report version.

Not only is the triarchic model bringing unity to the psychopathy literature [16] but its associated measure (the 58 item Triarchic Psychopathy Measure (TriPM) [21]) has the advantage that it focuses on the behavioural indicators of multidimensional psychopathy models and specifically includes the experiential aspects of psychopathy [5]. It indexes the three traits of boldness, meanness and disinhibition to the same degree and is appropriate for use with a general population rather than forensic or clinical applications. This makes it particularly suitable for self-report as well as other (non-expert) report.

The ability to gauge psychopathy in terms of self-reported traits and as other-perceived traits will enable multi-level organisational research, allowing the investigation of both the within-person effects of psychopathy and the impact of perceived psychopathic traits on co-workers. In this paper, therefore, we develop the TriPM (Work): a short, work-focused measure of psychopathy based on the triarchic model that can be used for both self- and other-report.

Using this measure, we test the proposition that boldness represents a predominantly adaptive domain of psychopathy while meanness and disinhibition capture the more maladaptive domains. We do this in three ways. First, we test the relationship between managers’ psychopathic traits and their leadership styles, as reported by subordinates. Second, we test the within-person effects of psychopathic traits on individuals’ work-related outcomes, such as well-being and performance; and third, we test the effects of managers’ perceived psychopathic traits on their subordinates’ work-related outcomes.

### 1.2. Psychopathy and Leadership Styles

Psychopathy in leaders is of particular concern given the potential of those in power to have significant negative influence on their followers and the ease with which decision-makers may “mistake psychopathic traits for specific leadership traits” [9] and promote more psychopathic individuals to leadership positions. There are many theories and models of leadership, and in order to distinguish the adaptive and maladaptive effects of psychopathy at work, we focus here on two leadership styles which represent contrasting approaches to leading others. Servant leadership is an other-oriented, beneficial style that has been shown to have incremental predictive validity over other beneficial forms of leadership such as ethical or transformational [22]. Abusive supervision, as an active form of destructive leadership, provides a distinct contrast and has strong negative effects on employee well-being and performance [23].

Servant leadership has a focus on prioritizing followers’ needs and demonstrating a concern for the wider organisation and community [24]. The relationship between psychopathy and servant leadership has not yet been addressed, but we would expect the traits of meanness and disinhibition, with their disregard of others, to be negatively related to servant leadership. We also suggest that boldness represents a trait essential to effective leadership and can manifest in an adaptive way at work and therefore would be positively related to this effective leadership style.

Abusive supervision is a distinctly contrasting leadership style that includes sustained, “hostile verbal and nonverbal behaviours, excluding physical contact” [25] by supervisors towards their subordinates. Recent research with employees working for a non-profit organisation who reported on their supervisors, demonstrated a positive relationship between psychopathy (using the B-Scan 360) and abusive supervision [26]. While findings such as these indicate some relationships between psychopathy and abusive supervision, research on the constituent dimensions of the triarchic model has not yet been conducted. We argue here that boldness is an adaptive and positive trait for a leader and will therefore be negatively associated with abusive supervision. In contrast, meanness and disinhibition, which index hostility towards others and a greater likelihood of engaging in abusive behaviour, respectively, are expected to be positively associated with abusive supervision.

### 1.3. Effects of Psychopathy at Work

Psychopathy is known to have both within- and between-person effects on employee well-being (including burnout and engagement) and job performance, but research findings so far show some inconsistencies. The triarchic model may provide insight into these contradictory findings, as the majority of occupationally based research uses overall measures of psychopathy rather than distinguishing between the effects of different psychopathic traits. We propose that the Job Demands-Resources (JD-R) model [27] can be used to explain the differential effects of the triarchic traits on these outcomes.

#### 1.3.1. Within-Person Effects

Research is starting to demonstrate that higher levels of psychopathy are associated with worse within-person well-being. For example, undergraduate students’ self-reported psychopathy was associated with lower levels of life satisfaction and happiness, as well as decreased environmental mastery, personal growth, positive relations with others, purpose in life and self-acceptance [28]. Similarly, Love and Holder [29] found psychopathy to be negatively related to life satisfaction, happiness and positive affect but positively related to both negative affect and depression. These findings reflect the somewhat contradictory nature of psychopathy [20]: despite psychopathy being defined as involving emotional detachment, higher levels of psychopathy are also associated with increased negative emotionality. The triarchic model captures this contradiction in the disinhibition trait [7].

Employee well-being is often operationalised in terms of burnout and engagement [30]. Burnout has been defined as a “prolonged response to chronic emotional and interpersonal stressors on the job, and is defined by the three dimensions of exhaustion, cynicism, and inefficacy” [31]. Although burnout is considered to be a consequence of stressors, it is also affected by individual characteristics such as psychopathic traits: Employees working full-time who self-reported higher levels of psychopathy also experienced greater emotional exhaustion [32]. Work engagement, on the other hand, refers to “a positive, fulfilling, work-related state of mind that is characterised by vigour, dedication, and absorption” [33]. Engagement is considered the positive antithesis to burnout [31], so while there is not, to the authors’ knowledge, any research directly investigating the effect of psychopathy on work engagement, we would expect that lower overall levels of psychopathy are related to higher engagement.

Meta-analysis has shown a negative relationship between overall psychopathy and job performance, although the effect size was small [34]. Employees with higher psychopathy scores are perceived to be poor team players and to lack management skills but are also seen as creative, innovative and good at communicating and thinking strategically [9], indicating that this overall relationship may be dependent on the constituent traits of psychopathy. For example, one study found that fearless dominance in US presidents, as rated by experts, was associated with higher job performance [35]. However, a more direct study where managers rated their own psychopathy and their superiors rated their job performance, found both no relationship between boldness and performance and a negative relationship between meanness/disinhibition and performance [36]. Other recent work has found a negative relationship between psychopathy and job performance [37] as well as both objective and subjective career success [38].

The conflicting effects of psychopathy on these work outcomes may be better understood by considering the adaptive and maladaptive elements of psychopathy as captured in the triarchic model.

#### 1.3.2. Between-Person Effects

The between-person effects of psychopathy on well-being mirror the findings of within-person effects, negatively affecting employee well-being. Managers who reported that a psychopathic individual was present in the workplace felt more angry, anxious, bored, depressed and discouraged, as well as less at ease, calm and content [39]. Furthermore, employees who rate their supervisors as more psychopathic report greater levels of psychological distress [17]. The impact of supervisor psychopathy on subordinate burnout has also been acknowledged in the literature, with subordinates who rate their direct supervisor as having higher levels of psychopathy reporting higher levels of emotional exhaustion [40].

As yet, there is no research investigating the effect of manager psychopathy on subordinate engagement or job performance.

#### 1.3.3. The Job Demands-Resources Model as a Framework

While this evidence demonstrates that psychopathy can be both adaptive and maladaptive at work [5], a model that could explain the mechanism of these occasionally inconsistent effects has not yet been proposed. We suggest that psychopathic traits can be conceptualised as demands or resources for workers and the Job Demands-Resources (JD-R) model [27] used to elucidate the adaptive and maladaptive effects of psychopathy on work outcomes. The JD-R distinguishes between job demands, which are aspects of the job requiring effort or skills and associated with physical or psychological costs, and job resources, which help workers achieve their goals, reduce job demands or stimulate development. It also recognises that personal characteristics, such as personality traits, play a role in how people interpret and respond to job demands.

In terms of within-person effects, we propose that the boldness trait, capturing social assertiveness, confidence and venturesomeness, is adaptive in that it enables the individual to access greater resources in response to job demands and perceive these demands more positively (as challenges rather than insurmountable problems). In contrast, the maladaptive traits of meanness and disinhibition result in poorer interpersonal relationships, so that individuals are less likely to be able to draw on social support (a job resource) in order to meet demands.

The JD-R model also suggests how the between-person effects of psychopathy may manifest at work. Boldness in a manager could act to provide greater access to resources for their subordinates and to present demands as positive challenges. On the other hand, interpersonal mistreatment at work is a job demand [41], and we therefore conceptualise the manager’s meanness and disinhibition as job demands for subordinates.

Job resources are associated with lower burnout and higher engagement, well-being and performance, while job demands are associated with higher burnout and lower engagement, well-being and performance [42]. As a job resource, boldness is therefore hypothesised to have a negative effect on burnout and a positive effect on engagement, well-being and job performance. In contrast, as job demands, meanness and disinhibition are hypothesised to have positive effects on burnout and negative effects on engagement, well-being and job performance.

In summary, by developing a measure of psychopathy in the workplace based on the triarchic model of psychopathy we expect to be able to refine our understanding of the adaptive and maladaptive elements of psychopathy at work. Boldness is expected to associate positively with servant leadership and negatively with abusive supervision, while the opposite should hold for meanness and disinhibition. In developing equivalent self- and other-report versions of this workplace measure, we will also be able to assess within-person effects and impacts on others. Boldness is expected to act as a job resource and have a positive effect both on the individual and subordinate’s work outcomes, while meanness and disinhibition are expected to act as job demands and result in negative effects.

## 2. Method

### 2.1. Design

Quantitative, two-phase data was collected from two different samples in New Zealand. Respondents completed an online survey at two time points, four weeks apart. Participants’ time 1 (T1) and time 2 (T2) responses were matched using personal IDs generated by the survey software. The T2 survey was kept open until a minimum of 300 participants had completed it.

Sample 1 consisted of managers reporting on their own psychopathy (amended TriPM, described below) and work-related outcomes (well-being, engagement, burnout and performance) and was used to test the within-person effects. Sample 2 consisted of employees reporting on their managers’ psychopathy (Note: the two samples were not linked. The managers in sample 1 were not reported on by employees in sample 2. Research on the consistency of self- and other perception of psychopathy is limited, and as our concern here was for developing equivalent measures rather than demonstrating consistency or difference between self-perceptions and follower perceptions of psychopathy, we did not use matched samples.) (amended TriPM), servant leadership and abusive leadership as well as self-report measures of their own work-related outcomes (well-being, engagement, burnout and performance) and was used to test the between-person effects and the relationships between perceived psychopathy and leadership styles.

### 2.2. Participants

Sample 1 consisted of 679 managers at time 1 (T1) and 300 at time 2 (T2), a 44.2% retention rate. At T1, the sample was 43% female, 56% male and 1% identified as another gender or preferred to not state. The mean age was 42.7 years (SD = 13.2) and mean job tenure 7.45 years. No information on ethnicity was collected. Largest industry categories were retail, trade and accommodation (14.3%) and education and training (12.3%). Slightly more female than male respondents were retained in the T2 sample (46% female) and the mean age increased to 46.5 years (SD = 21.5), with a similar proportion of industry sectors as at T1.

Sample 2 consisted of 697 employees at T1 and 331 at T2, a 47.5% retention rate. At T1, the sample was 52% female and 48% male, with a mean age of 38 years (SD = 12.6) and mean job tenure of 5.83 years. Many respondents worked in retail, trade and accommodation (15.2%) and in health care and social assistance (14.8%). Similarly to the manager sample, more female than male respondents were retained at T2 (58% female), and the mean age increased to 40.48 years (SD = 12.5) with a similar spread of industries as at T1.

### 2.3. Measures

#### 2.3.1. Demographics

The following demographic characteristics were collected at T1: age, gender, tenure in current job and industry sector (using the Australian and New Zealand Standard Industrial Classification (ANZSIC) 2006 categories). In addition, the manager sample reported their number of direct reports and any formal leadership training (5 categories: Undergraduate university qualification (e.g., BA Management), Postgraduate university qualification (e.g., MBA), In-house training, Formal mentorship program or Other).

#### 2.3.2. Psychopathy

Self-reported psychopathy was assessed with the TriPM, a 58-item measure of the triarchic psychopathy model, adapted as described below to make it applicable to a workplace setting. The scale consists of three dimensions characteristic of psychopathy, namely boldness, meanness and disinhibition, and items are rated on a 4-point scale (1 = false, 4 = true). Cronbach alphas are reported as 0.79 (Boldness), 0.83 (Meanness) and 0.79 (Disinhibition) [43]. Item wordings are available at the PhenX website [44] and will be referred to by number here. For this and all following measures, unless otherwise noted, the mean score was calculated for each scale and used in further analysis.

Items were screened for face validity and suitability for administration to a workplace sample. Eight items (#4, 5, 14, 18, 24, 43, 53, 58) which had low face validity for the workplace (e.g., I have no strong desire to parachute out of an airplane) or would require knowledge of the manager, which the subordinate might not have (e.g., (My manager) has gotten in trouble because s/he missed too much school) were removed. In order to develop an equivalent other-report version of the TriPM, “I” was changed to “my manager” for all items and some rewording was necessary to maintain the meaning of the item. For example, “Others have told me they are concerned about my lack of self-control” was changed to “People are concerned about my manager’s lack of self-control”. The amended measure consisted of 50 items for both self- and other-report.

#### 2.3.3. Servant Leadership

The servant leadership measure [45] consists of 14 items rated on a 5-point Likert scale (1 = to a small extent, 5 = to a very large extent). A sample item is “My manager creates a sense of community among employees”. The original wording of “department manager” was changed to “manager” for this study. Internal consistency for this scale is reported as 0.98 [45].

#### 2.3.4. Abusive Supervision

Abusive supervisory behaviours were measured using the Tepper [25] scale, with the original wording of “boss” changed to “manager” for consistency with other scales. The scale consists of 15 statements rated on a 5-point Likert scale (1 = I cannot remember my manager ever using this behaviour with me, 5 = Uses this behaviour with me very often). A sample item is “Reminds me of my past mistakes and failures”. Reported Cronbach alpha for this scale is 0.9 [25].

#### 2.3.5. Burnout

The abbreviated Maslach Burnout scale [46] was used to assess burnout. Again, in line with the literature, we utilise an overall burnout score rather than the three subscales of emotional exhaustion, depersonalisation, and professional efficacy [46]. In order to make the scale applicable to the workplace, the word “patients” has been changed to “people” in the respective statements. The measure consists of nine items, for example, “I feel I treat some people at work as if they were impersonal objects”, rated on a 7-point Likert scale (1 = never; 7 = everyday).

#### 2.3.6. Engagement

Engagement was assessed with the short version of the Utrecht Work Engagement Scale (UWES; [47]). It is a 9-item measure consisting of the three dimensions of vigour, dedication and absorption, though we follow recommendations in the literature and combine all items into a single measure of engagement [48]. Items are rated on a 7-point Likert scale (1 = never; 7 = always (every day)). A sample item is “At my work, I feel bursting with energy”.

#### 2.3.7. Well-Being

Well-being was assessed with the Warwick–Edinburgh Mental Well-being Scale (WEMWBS; [49]). The WEMWBS is a unidimensional measure that consists of 14 items and contains statements that refer to hedonic as well as eudaimonic well-being and can be rated on a 5-point Likert scale (1 = none of the time; 5 = all the time). A sample item is “I’ve been feeling cheerful”. The WEMWBS has high internal consistency, with Cronbach alpha of 0.89 for a student sample and 0.91 for a general population sample, and good test-retest reliability (0.83 over one week) [49].

#### 2.3.8. Performance

Performance was assessed using three items from the World Health Organisation Health and Work Performance Questionnaire (HPQ) [50]. The first two items act as internal anchors by asking the respondent to rate the performance of the average worker doing their job and then their own usual performance before rating the final item: “Your own overall job performance on the days you have worked during the past 6 months” [50]. The rating scale is 1–10, (where 1 = the worst performance anyone could have at your job, 5 to 6 = average level of performance and 10 = the performance of a top worker). The final item is used as a global index of subjective job performance, and the HPQ shows good concordance with objective performance measures.

### 2.4. Data Cleaning

Cases with >10% missing values were removed. Outliers were removed based on a combination of the Mahalanobis distance, used to identify multivariate outliers [51], and the participants’ response time. Mahalanobis distance was calculated for each case based on all items from the TriPM measure and compared to a Chi-square distribution with the same degrees of freedom (df = 49). As advised by Tabachnick and Fidell [51], a very conservative probability estimate of *p* < 0.001 was used to identify potential outliers. In addition, a response time faster than 50% of the median time [52] was taken as an indication that the participants may not have given quality responses. Therefore, cases with both a significant Mahalanobis distance as well as a fast response time were removed from the data sets. This resulted in a final sample of 651 managers and 668 employees at T1 and 286 managers and 318 employees at T2.

### 2.5. Data Analysis

The development of short self- and other-report TriPM scales applicable to the workplace was addressed by reducing the number of items in each scale while maximizing internal consistency, evaluating convergent validity with the original scales, and checking test-retest reliability.

The TriPM scales were originally drawn from large multi-scale inventories and developed to optimise both content representation and accurate scoring. While the TriPM captures a substantial amount of the variance in other psychopathy measures [53], and there is some support from SEM for the three factor structure [5], the TriPM does not measure the three components as distinct factors but rather as inter-related phenotypic constructs [3]. Because of this, we do not expect CFA to identify three distinct factors in the TriPM and did not subject the whole measure to CFA. Instead, in order to identify the items that most strongly represent each subscale in a working population, we conducted principal component analysis on each subscale in turn, forcing one factor and ranking items according to their highest loadings. From the top ten highest loading items for each scale, we selected 7 items that were identical across self- and other-report to create a short, 21-item measure of the triarchic model of psychopathy at work (TriPM (Work)).

Internal consistency of the TriPM (Work) scales was evaluated with Cronbach alpha. Their convergent validity with the original scales, as well as the internal relationships between the scales, was evaluated using Pearson correlations. Finally, test–retest reliability was assessed by calculating Pearson correlations between the T1 and T2 data.

Hypotheses about the relationship between other-reported psychopathy and leadership style were also assessed using Pearson correlations at T1. Subsequently, hypotheses of the differential effects of psychopathic traits at T1 on work outcomes at T2 (within- and between-person effects) were tested using regression analyses. We use *p* < 0.05 as the target significance level for all statistical analyses and report effect sizes where appropriate.

## 3. Results

### 3.1. The TriPM(Work)

As described in Section 2.5, we conducted CFA on each TriPM scale and selected the 7 highest-loading items that were identical across self- and other-report to create a 21-item TriPM (Work) measure, with the same seven items per scale. Items retained for the TriPM(Work) boldness scale are 7, 10, 13, 16, 22, 50, 57; for the meanness scale 9, 12, 15, 37, 49, 51, 56 and for the disinhibition scale 20, 23, 26, 29, 36, 48, 55.

The correlations between the full TriPM and their equivalent TriPM(Work) scales were all above 0.9, indicating a close to perfect relationship and demonstrating convergent validity. Cronbach’s alphas for TriPM(Work) scales were above 0.7 (see Table 1 and Table 2) indicating adequate internal consistency. The correlations between the self-report TriPM(Work) scales generally conform to those found in studies using the full TriPM [4], with moderate positive correlations between disinhibition and meanness, although the boldness scale shows somewhat stronger negative correlations with the other two subscales than in previous work.

Using matched T1 and T2 data, test–retest reliability after 4 weeks was evaluated. All scales demonstrated adequate test-retest reliability, with T1-T2 correlations above 0.70, as shown in Table 3.

### 3.2. Psychopathy and Leadership Styles

Subordinate perceptions of managers’ boldness were expected to correlate negatively with abusive leadership and positively with servant leadership. These relationships were supported (see Table 2). The opposite pattern of results was expected for the meanness and disinhibition scales and again was confirmed.

### 3.3. Effects of Psychopathy at Work

Using the JD-R model, we predicted that boldness, as a job resource, would relate positively to engagement, well-being and job performance and negatively to burnout, while meanness and disinhibition, as job demands, are expected to show the opposite pattern of results. Matched T1 and T2 data (see Table 4 and Table 5) was used to test these predictive relationships, with regression analyses conducted using TriPM(Work) scales at T1 predicting outcomes at T2.

#### 3.3.1. Within-Person Effects

Self-reported psychopathy predicted a substantial amount of variance in well-being (31%), engagement (23%), burnout (33%) and performance (12%) (see Table 6). Boldness predicted increased well-being (*ß* = 0.56, t(281) = 10.65, *p* < 0.001); engagement (*ß* = 0.43, t(281) = 7.68, *p* < 0.001) and performance (*ß* = 0.32, t(279) = 5.31, *p* < 0.001) along with reduced burnout (*ß* = −0.33, t(280) = −6.36, *p* < 0.001). The expected negative effect of meanness on engagement did not reach significance, but there was a positive effect on burnout (*ß* = 0.26, t(281) = 4.11, *p* < 0.001). Similarly, disinhibition increased (burnout *ß* = 0.16, t(281) = 2.46, *p* < 0.01) but had no significant effect on other outcomes.

#### 3.3.2. Between-Person Effects

Managers’ perceived psychopathy did not significantly predict subordinate well-being or performance and perceived boldness, and disinhibition did not have the expected effects on any of the employee outcomes (see Table 7). However, managers’ meanness did predict reduced subordinate engagement (*ß* = −0.18, t(314) = −1.98, *p* < 0.05) and increased burnout (*ß* = 0.25, t(313) = 2.79, *p* < 0.01).

In summary, managers’ self-reported psychopathy predicts a substantial amount of variance in all four work outcomes, with boldness showing the predicted positive effects and meanness and disinhibition both increasing burnout. When measured by other-report, manager’s meanness predicts reduced subordinate engagement and increased burnout.

## 4. Discussion

Overall, we find that the TriPM (Work) provides a reliable and valid measure of psychopathy at work and can be used for both self- and other-report. Similarly to the original TriPM measure, the three subscales of the TriPM (Work) have good internal consistency and test–retest reliability. The scales also demonstrate the expected relationships with several work variables. The boldness trait, largely representing the adaptive elements of psychopathy at work, shows positive relationships with a beneficial leadership style and positive work outcomes for the individual. Meanness and disinhibition traits, representing the maladaptive elements of psychopathy, are associated with a poor leadership style and negative work outcomes for the individual and, in the case of meanness, for subordinates as well.

The pattern of results between psychopathic traits and work outcomes is consistent with our proposition that boldness may act as a resource within the JD-R model and improve engagement, well-being and job performance, while meanness and disinhibition act to increase demands and thereby burnout. Whether this is a direct effect of the traits or whether the effect is mediated by other factors such as perception of job demands or influence on social support remains for future research to explore. A recent study indicated that subordinates’ psychopathy may serve to maintain personal resources in the face of abusive supervision [54], though as it did not use the triarchic model, it is unclear whether this is due primarily to the boldness trait acting as a resource or a high level of overall psychopathy. Clearly, understanding the intricacies of the adaptive effects of psychopathy requires detailed examination of these relationships, and it is our hope that the TriPM (Work) measure we report on here will support future research in this area.

One of the most striking findings of this study is that psychopathic traits have larger within-person than other-person effects. This provides a contrast to much of the literature around psychopathy at work, which emphasises the negative impact of psychopathy on others in the workplace (e.g., [6]). We found that, as hypothesised, managers’ self-reported boldness has a large positive effect on their work outcomes (increasing well-being, engagement and performance and reducing burnout) while meanness and disinhibition both increase burnout. Nevertheless, the effects of managers’ perceived psychopathy on subordinates are not as substantial as these within-person effects. In addition, the effect of managers’ psychopathy on subordinates seems to be limited to a small negative impact from the meanness trait, with no positive effect of boldness on subordinates’ work outcomes. This finding raises important questions for our understanding of psychopathy at work, and the distinction between primary and secondary psychopathy may shed some light on this issue. Corporate psychopathy has been described as including the callous emotional traits associated with primary psychopathy but not exhibiting the same level of anti-social or criminal tendencies of secondary psychopathy [6]. Our findings may provide some support for this distinction by demonstrating that psychopathy at work is associated with a substantial effect on the individual’s own emotional well-being and engagement and less effect, via anti-social behaviour, on those around them.

The largest effect sizes in our study were the relationships between the triarchic traits and leadership styles, which are substantially larger than those found in a self-report study of psychopathy and the full-range leadership model [55]. Besides the differences in instruments used to assess psychopathy and leadership, there are two potential explanations for this finding. First, it may indicate that the TriPM (Work) is tapping into behaviours that are seen as particularly salient to employees’ perceptions of leadership style. With correlations above 0.7 for the relationship between abusive supervision and both meanness/disinhibition, for example, it appears that these maladaptive psychopathy traits may be almost synonymous with destructive leadership. Alternatively, the correlations may be subject to a negative halo effect. Previous work on supervisor personality and bullying has found that employees rate their supervisors’ personality as significantly less socially desirable when they observe or experience bullying at work [56]. We discuss this further in Section 4.2.

These relationships do, however, confirm the suggestion that psychopathic traits are related to the leadership styles that managers adopt. Again, boldness was confirmed as an adaptive element of psychopathy, related to greater servant leadership and lower abusive supervision while meanness and disinhibition were maladaptive, being related to less servant leadership and greater abusive supervision. To our knowledge, this is the first paper to report on the relationship between servant leadership and psychopathy, and it demonstrates the utility of using a triarchic model to measure psychopathy. A positive relationship between psychopathy and servant leadership would appear at first glance to be highly unlikely, yet decades of research support the relationship between effective leadership and social assertiveness or confidence [57]. It seems that the boldness element of the triarchic model of psychopathy captures this essential leadership quality and may underlie at least some of the success of more highly psychopathic individuals at work.

We noted in the introduction that researchers have suggested boldness should not be considered part of the psychopathy concept precisely because of these kinds of adaptive outcomes [11]. The meta-analytic review that confirmed the relevance of boldness to psychopathy noted that boldness was likely to be substantially more important for the study of psychopathy in community rather than forensic samples, where the emphasis is on adaptive functioning [15]. The authors also caution against assuming that psychopathy, as measured in community settings, is exactly the same construct as measured in forensic settings. In this paper, we have focused on delineating the adaptive and maladaptive effects of psychopathic traits at work, demonstrating that boldness represents an adaptive element of the overall psychopathy construct while meanness and disinhibition are maladaptive.

### 4.1. Implications

This study demonstrates that the TriPM (Work) is a reliable and valid measure of the triarchic model of psychopathy for the workplace. With only 21 items, it is well suited to organisational research where participants’ time is frequently limited, while equivalent self- and other-report versions promise to make comparative and multi-level research simpler.

In addition, we have demonstrated that the triarchic model of psychopathy is useful in distinguishing between the adaptive and maladaptive effects of psychopathy at work. As suggested by previous authors (e.g., [7]), boldness can indeed be viewed as an adaptive element of psychopathy while meanness and disinhibition are maladaptive in the workplace. The JD-R model provides a way of conceptualizing these distinctive effects as resources or demands for both the individual and their co-workers.

The finding that psychopathy has a greater effect on managers themselves than on their subordinates suggests that interventions aimed at reducing the negative effect of psychopathy in the workplace should focus on self-development rather than attempting to buffer or reduce the effect of “psychopathic leaders” on their subordinates. It was recognised early on that psychopathy, even in noninstitutionalised populations, includes a distinctly self-defeating lifestyle [58], and this study demonstrates that meanness and disinhibition in particular have significant negative within-person effects. Using the regression equations reported here, for example, the average manager’s boldness score predicts very low burnout (1.8 on a 7-point scale) and high engagement (5.6 on a 7-point scale), while average meanness and disinhibition predict much higher burnout (4.9 and 4.5 on a 7-point scale). Interventions aimed at helping managers to increase their boldness and reduce meanness/disinhibition could therefore have substantial positive workplace effects.

### 4.2. Limitations and Further Research

It still remains to be determined what level of psychopathic traits would be considered necessary and sufficient to categorise someone as a “psychopath” at work. While some measures in the clinical and forensic field utilise cut-off scores [7], we have avoided drawing a cut-off here as we were interested in psychopathic traits as continuous dimensions in the normal population. Future research could explore the extent to which different trait profile patterns may be associated with adaptive and maladaptive outcomes. For example, is the adaptive effect of boldness weakened when combined with higher meanness or disinhibition scores?

We note that the inter-correlations between the other-report TriPM (Work) scales are higher than has been found in studies using the full self-report TriPM [4]. This may be due to a halo effect, which is an established issue in leadership research: if a leader is seen as effective, they are rated more positively across a range of categories and vice versa [58].

We also acknowledge that the lack of a matched sample of employees and managers means that we are unable to establish the extent to which the self- and other-report TriPM (Work) measures may correspond for the same individual. We expect there may be reasonable levels of concordance between self- and other-ratings for two reasons. First, the TriPM (Work) items focus on observable behaviours rather than inference about internal states and is therefore reasonably straightforward for observers to report. Second, a recent meta-analysis of self- and other-reports across a range of personality questionnaires (collated into the Big Five traits) showed work colleagues’ reports on personality as not being significantly different from self-reports. It remains for future research to determine the extent to which this finding may be generalizable to psychopathic traits. However, concordance is not of itself essential for the use of these measures in future work. For example, the extent to which a manager’s self-reported psychopathy corresponds with subordinate’s perceptions may be used as the basis for 360 feedback, much as the B-Scan 360 aims to do [18].

While there is some evidence here that boldness may act as a resource and meanness/disinhibition as demands, in terms of their effects on engagement, burnout, well-being and performance, these relationships would benefit from further research. In particular, the mechanism of these effects is as yet unknown. For example, meanness could have its effect either by acting as an extra demand on the individual or via destruction of social support (i.e., removal of resources).

One of the strengths of this study is the time-lagged data, which enabled predictive relationships to be explored properly. However, as the time-lag was only 4 weeks, further research looking at the long-term effects of psychopathy would be valuable. Whether boldness, for example, would continue to have positive effects over the longer-term is unknown. It may also be that a longer-term study would also be able to identify greater effects of psychopathy on subordinates.

## 5. Conclusions

The triarchic model of psychopathy is useful in distinguishing between adaptive and maladaptive psychopathic traits in the work context and the TriPM (Work) scales provide a reliable, organisationally-relevant measure for both self- and other-report. Boldness is associated with primarily positive leadership styles and work outcomes, while meanness and disinhibition are associated with poor leadership styles and negative work outcomes. Additionally, psychopathic traits are associated with much greater within-person than cross-person effects.

## Figures and Tables

**Table 1:** Descriptive statistics for Sample 1 (managers) at Time 1 (*N* = 651).

Variables	Min	Max	M	SD	1	2	3	4	5	6
1. Boldness	1.00	4.00	2.93	0.46	(0.74)					
2. Meanness	1.00	4.00	1.69	0.61	−0.29 **	(0.90)				
3. Disinhibition	1.00	4.00	1.73	0.65	−0.33 **	0.64 **	(0.87)			
4. Well-being	1.21	5.00	3.51	0.63	0.48 **	−0.13 **	−0.21 **	(0.93)		
5. Engagement	1.00	7.00	5.10	1.21	0.42 **	−0.19 **	−0.18 **	0.59 **	(0.94)	
6. Burnout	1.00	6.33	3.01	1.01	−0.48 **	0.44 **	0.48 **	−0.47 **	−0.57 **	(0.77)
7. Performance	3.00	10.0	7.80	1.45	0.29 **	−0.19 **	−0.23 **	0.43 **	0.46 **	−0.37 **

Note. Min and Max represent the minimum and maximum scores on each scale reported by participants. M = mean score, SD = standard deviation. Alpha reliabilities in parentheses. ** *p* < 0.01 (two-tailed).

**Table 2:** Descriptive statistics for Sample 2 (employees) at Time 1 (*N* = 668).

Variables	Min	Max	M	SD	1	2	3	4	5	6	7	9	10
1. Boldness	1.00	4.00	2.92	0.66	(0.86)								
2. Meanness	1.00	4.00	1.85	0.80	−0.63 **	(0.94)							
3. Disinhibition	1.00	4.00	1.70	0.70	−0.64 **	0.76 **	(0.90)						
4. Well-being	1.00	5.00	3.33	0.66	0.14 **	−0.15 **	−0.14 **	(0.93)					
5. Engagement	1.00	7.00	4.73	1.30	0.26 **	−0.28 **	−0.19 **	0.52 **	(0.94)				
6. Burnout	1.00	6.33	3.27	1.04	−0.33 **	0.39 **	0.33 **	−0.49 **	−0.61 **	(0.75)			
7. Performance	1.00	10.0	7.53	1.59	0.12 **	−0.15 **	−0.17 **	0.36 **	0.33 **	−0.34 **			
8. Servant	1.00	5.00	3.00	1.00	0.67 **	−0.58 **	−0.47 **	0.24 **	0.36 **	−0.30 **	0.14 **	(0.96)	
9. Abusive	1.00	4.87	1.70	0.88	−0.58 **	0.77 **	0.71 **	−0.18 **	−0.27 **	0.42 **	−0.21 **	−0.43 **	(0.97)

Note. Min and Max represent the minimum and maximum scores on each scale reported by participants. M = mean score, SD = standard deviation. Alpha reliabilities in parentheses. ** *p* < 0.01 (two-tailed).

**Table 3:** TriPM(Work) test–retest reliability (Time 1 and Time 2 Pearson correlations).

Variables	Sample 1 (Self-Report)	Sample 2 (Other-Report)
Boldness	0.74 **	0.77 **
Meanness	0.76 **	0.72 **
Disinhibition	0.73 **	0.78 **

Note. ** *p* < 0.01 (two-tailed).

**Table 4:** Descriptive statistics for Sample 1 (managers, matched T1 and T2 data) (*N* = 286).

Variables	M	SD	1	2	3	4	5	6
1. Boldness T1	2.98	0.44	(0.70)					
2. Meanness T1	1.68	0.59	−0.29 **	(0.89)				
3. Disinhibition T1	1.68	0.63	−0.34 **	0.62 **	(0.88)			
4. Well-being T2	3.52	0.63	0.55 **	−0.17 **	−0.13 *	(0.92)		
5. Engagement T2	5.18	1.11	0.47 **	−0.25 **	−0.22 **	0.55 **	(0.94)	
6. Burnout T2	2.99	1.03	−0.46 **	0.45 **	0.43 **	−0.42 **	−0.61 **	(0.77)
7. Performance T2	7.91	1.41	0.33 **	−0.16 **	−0.13 *	0.47 **	0.51 **	−0.33 **

Note. * *p* < 0.05 (two-tailed), ** *p* < 0.01 (two-tailed).

**Table 5:** Descriptive statistics for Sample 2 (employees, matched Time 1 and Time 2 data) (*N* = 318).

Variables	M	SD	1	2	3	4	5	6
1. Boldness T1	2.94	0.65	(0.87)					
2. Meanness T1	1.82	0.78	−0.63 **	(0.94)				
3. Disinhibition T1	1.69	0.71	−0.66 **	0.77 **	(0.91)			
4. Well-being T2	3.30	0.68	0.03	−0.09	−0.10	(0.94)		
5. Engagement T2	4.81	1.30	0.16 **	−0.19 **	−0.16 **	0.55 **	(0.95)	
6. Burnout T2	3.21	1.06	−0.20 **	0.30 **	0.27 **	−0.45 **	−0.62 **	(0.76)
7. Performance T2	7.52	1.64	0.07	−0.13 *	−0.16 **	0.41 **	0.40 **	−0.37 **

Note. Alpha reliabilities in parentheses. * *p* < 0.05 (two-tailed), ** *p* < 0.01 (two-tailed).

**Table 6:** Regression analyses of self-reported psychopathy (Time 1) predicting well-being, engagement, burnout and performance (Time 2).

Variables	Well-Being T2			Engagement T2			Burnout T2			Performance T2		
	*B*	*SE B*	*ß*	*B*	*SE B*	*ß*	*B*	*SE B*	*ß*	*B*	*SE B*	*ß*
Intercept	1.04 ***	0.28		2.32 ***	0.53		4.13 ***	0.45		5.08 ***	0.71	
Boldness T1	0.82	0.08	0.56 ***	1.09	0.14	0.43 ***	−0.78	0.12	−0.33 ***	1.03	0.19	0.32 ***
Meanness T1	−0.08	0.07	−0.08	−0.24	0.13	−0.13	0.45	0.11	0.26 ***	−0.23	0.17	−0.10
Disinhibition T1	0.11	0.07	0.11	0.00	0.12	0.00	0.26	0.10	0.16 **	0.09	0.17	0.04
*R* ^2^	0.31			0.23			0.33			0.12		
*F*	42.01 ***			28.17 ***			47.05 ***			12.30 ***		

Note. B = unstandardized beta value, SE B = standard error of beta value, *ß* = standardized beta. ** *p* < 0.01. *** *p* < 0.001.

**Table 7:** Regression analyses of perceived manager psychopathy (Time 1) predicting engagement, burnout and performance in subordinates (Time 2).

Variables	Well-Being T2			Engagement T2			Burnout T2			Performance T2		
	*B*	*SE B*	*ß*	*B*	*SE B*	*ß*	*B*	*SE B*	*ß*	*B*	*SE B*	*ß*
Intercept	3.78 ***			4.96 ***	0.65		2.27 ***	0.52		8.85 ***	0.83	
Boldness T1	−0.08	0.08	−0.08	0.12	0.15	0.06	0.03	0.12	0.02	−0.17	0.20	−0.07
Meanness T1	−0.04	0.08	−0.05	−0.30	0.15	−0.18 *	0.34	0.12	0.25 **	−0.09	0.19	−0.04
Disinhibition T1	−0.11	0.09	−0.12	−0.03	0.17	−0.02	0.13	0.14	0.09	−0.40	0.22	−0.17
*R* ^2^	0.01			0.04			0.10			0.03		
*F*	1.41			4.76 **			11.06 ***			3.00 *		

Note. B = unstandardized beta value, SE B = standard error of beta value, *ß* = standardized beta. *p < 0.05. ** *p* < 0.01. *** *p* < 0.001.
